# Intraretinal calcium channels and retinal morbidity in experimental retinopathy of prematurity

**Published:** 2011-09-27

**Authors:** Bruce A. Berkowitz, David Bissig, Deborah Bergman, Emanuela Bercea, Vijaya K. Kasturi, Robin Roberts

**Affiliations:** 1 Department of Anatomy and Cell Biology, Wayne State University, Detroit, MI; 2 Department of Ophthalmology, Wayne State University, Detroit, MI

## Abstract

**Purpose:**

To test the hypothesis that intraretinal calcium channels participate in retinal morbidity in a variable oxygen (VO) model of retinopathy of prematurity.

**Methods:**

In control and VO Long Evans (LE) rats, either untreated or treated with voltage- or ligand-gated calcium channel antagonists, we measured retinal neovascular (NV) incidence and severity (adenosine diphosphatase staining), and retinal thickness and intraretinal ion channel activity (manganese-enhanced magnetic resonance imaging). Comparisons with the commonly studied Sprague Dawley rats were performed. Visual performance (optokinetic tracking) in untreated VO LE rats was also evaluated.

**Results:**

In control LE rats, specific L-type voltage calcium channel antagonism, but not ligand-gated channel blockers, suppressed retinal manganese accumulation, while the inhibition of L-type channels normalized intraretinal uptake in VO LE rats. VO LE rats developed more severe NV than VO Sprague Dawley rats. Following VO, both strains demonstrated significant and similar degrees of retinal thinning and supernormal intraretinal manganese uptake. However, over time, intraretinal uptake remained elevated only in VO LE rats. Visual performance was subnormal in VO LE rats. L-type voltage-gated calcium channel antagonism reduced NV severity by 28% (p<0.05) in experimental LE rats compared to that in the control group.

**Conclusions:**

Abnormal intraretinal calcium channel activity is linked with retinal morbidity in experimental retinopathy of prematurity.

## Introduction

Retinopathy of prematurity (ROP) is the major sight-threatening complication of preterm birth, with infants of shorter gestation periods at higher risk for retinal and visual morbidity. Peripheral vasculature abnormalities, such as retinal neovascularization (NV), are a clinical hallmark of ROP, although evidence has accumulated that central retinal lesions that do not involve the circulation also participate in the pathology [1-6]. Acute vision loss linked with retinal NV (and subsequent retinal detachment) does not develop in all preterm infants, and if NV does appear, it often resolves spontaneously. Yet patients with a history of ROP can demonstrate life-long injury to retinal structure [4,7] and function, as well as impaired vision [1,8]. Current retinal ablative treatments are primarily focused on reducing retinal NV, but these are destructive and only partially effective.

At present, the molecular mechanisms underlying retinal NV, as well as lesions to the central retinal structure (e.g., retinal thinning found in experiment models in vivo and ex vivo) and function linked with ROP, are insufficient to serve as a basis for effective targeted drug treatment. Growing evidence has raised the possibility that abnormal calcium channel activity contributes to retinal morbidity in ROP [4,9-11]. In a variable oxygen (VO) exposure model of ROP, examination of light-adapted rat retinas in vivo with manganese-enhanced magnetic resonance imaging (MEMRI) demonstrated retinal thinning and supernormal intraretinal manganese uptake consistent with increased calcium access [4]. Voltage-gated calcium channels are a major entry point for calcium and manganese into the cytoplasm [12-14]. Importantly, antagonism of L-type voltage-gated calcium channels in rodent NV models significantly reduced retinal NV severity [9-11]. These considerations led to the hypothesis that intraretinal calcium channels participate in retinal morbidity in experimental ROP.

In this study, we further test this hypothesis in a clinically relevant VO rat model [15] using a combination of specific calcium channel antagonists, noninvasive structural and functional (MEMRI) tests in vivo, and histologic evaluation ex vivo. To better understand the importance of early structural and functional lesions, two rat strains were examined and outcomes compared. MEMRI provides a spatially accurate measure of whole retinal thickness in vivo colocalized with measures of intraretinal function based on the intraretinal uptake of manganese, a calcium ion surrogate [16-18]. Furthermore, visual performance was assessed in the VO model using optokinetic tracking (OKT). OKT rapidly measures changes in rodent spatial frequency threshold and contrast sensitivity [19-22]. The OKT test does not require rodent training, is highly reproducible, and is readily performed in very young and adult rats without fatiguing the animal [22].

## Methods

All animals were treated in accordance with the NIH Guide for the Care and Use of Laboratory Animals, the ARVO Statement on Animals in Vision research, and Institutional Animal and Care Use Committee authorization.

### Animal Groups

All rats were housed in the laboratory and maintained in a normal 12 h: 12 h light-dark cycle .

### Experiments involving room-air controls

Control (untreated or saline-injected) Long Evans (LE) or Sprague Dawley (SD) rats were raised in room air until either postnatal day (P)14, P19–21 (for simplicity, this group will be referred as “P20”), or P43–63 (“P50”); no selection for sex was made. These LE and SD rats were studied using MEMRI and wholemount analysis; a subset of LE P50 rats was studied by OKT. Note that the MEMRI data for these controls were the controls for the age-matched VO groups. To check that LE rats demonstrated the light/dark adaptation patterns on MEMRI examination, a small group of light-adapted LE P50 rats were also studied, as previously described [17,18,23]. Following overnight dark adaptation, rats were maintained in normal laboratory lighting conditions for 30 min before MnCl_2_ injection, and for the next 4 h before MEMRI examination.

Dark-adapted control male P50 LE rats were treated with combinations of specific antagonists to either L-type voltage-gated calcium channels (30 mg/kg nifedipine [NIF], dimethyl sulfoxide (DMSO), intraperitoneally [i.p.]) or ligand-type calcium channels (4 mg/kg MK-801, saline, i.p. [MK], and 10 mg/kg 2,3-dihydroxy-6-nitro-7-sulfamoyl-benzo[f]quinoxaline-2,3-dione (NBQX), saline, i.p. [NB]) 30 min before MnCl_2_ injection, and then studied only with MEMRI 4 h later; controls were injected with either DMSO or saline 30 min before administration of MnCl_2_ and MEMRI 4 h later. These antagonist studies were performed on unilaterally patched rats as part of an ongoing investigation of aging. Only data from the patched eyes were considered in this study, as they are most relevant to the present dark-adapted experiments. Intraretinal manganese uptakes in control male P50 LE rats between patched retinas and never-patched but dark-adapted retinas were not different (p>0.05), and were combined for further comparisons.

### Experiments involving untreated variable oxygen rats

The newborn VO rat model has been described in detail elsewhere [24,25]. Briefly, LE and SD dams and litters (12–15 pups per litter) were housed in a modified pediatric incubator in which the oxygen levels were varied between 50% and 10% (50/10) every 24 h until P14. Rats were then allowed to recover in room air for either 0 (i.e., 14/0 [P14]), 6 (14/6 [P20]), or 36 (14/36 [P50]) days; some rats received saline (subcutaneously [s.c.], between 7/0 and 14/6). All animals received normal rat chow. No selection for sex was made. The above LE and SD rats were studied using MEMRI and wholemount analysis; a subset of 14/36 LE rats was also studied by OKT. Note that the untreated SD VO results in this study were, as expected, similar to historical data but not derived from those data.

### Experiments involving treated variable oxygen rats

Treating VO LE pups with i.p. injections of NIF in DMSO (30 mg/kg) between 14/0 and 14/6 was fatal, so instead, we investigated the benzothiazepine calcium channel antagonist D-*cis*-diltiazem (DIL) because it is water soluble and a primary antagonist of L-type voltage-gated calcium channels in vivo [26-29]. In treatment *arm A*, DIL (30 mg/kg, s.c.) was administered to LE rats between 14/0 and 14/6. In *arm B*, LE rats were treated with a combination of diltiazem (30 mg/kg, s.c., between 7/0 and 14/6) and nifedipine (dam, chow admix, approximately 30 mg/kg/day, between 7/0 and 14/6) (DIL+NIF). These animals were studied using MEMRI and wholemount analysis.

### Wholemount analysis

Adenosine diphosphatase–stained wholemounts of all infant rats were analyzed, as previously described, to determine retinal NV incidence and severity [30,31]. To determine NV severity, two investigators independently scored each wholemount in clock hours (score: 0–12) of NV in a masked fashion, and for each retina, the median of these two scores was calculated. The use of such clock hour assessment in stained wholemounts to measure NV severity analytically has been validated against counting cell nuclei above the inner limiting membrane [32]. No selection for sex was made.

### Manganese-enhanced magnetic resonance imaging

In all cases, rats were maintained in darkness overnight and injected with MnCl_2_ the following day. In unpatched rats, all procedures (e.g., weighing, injecting MnCl_2_, anesthetic administration, and MRI exam) were done under dim red light or darkness. MnCl_2_ was administered as an i.p. injection (44 mg MnCl_2_·4H_2_O/kg) on the right side of each awake and free-moving rat. Unpatched rats were maintained in dark conditions for another 4 h, anesthetized using urethane (36% solution, i.p., 0.083 ml/20 g animal weight, prepared fresh daily, Sigma-Aldrich, Milwaukee, WI), and then examined by MEMRI. After the MEMRI examination, rats were killed with an intracardiac potassium chloride injection. ROP and age-matched control rats also had both eyes enucleated and retinas wholemounted for staining and NV analysis. No selection for sex was made.

All rats were gently positioned in a specialized rat cradle. MRI data were acquired on either a 4.7 T Bruker Avance (Sprague Dawley) or 7 T Bruker Clinscan (Long Evans) system using a surface coil (1.0 cm diameter) placed over the left eye. On the 4.7 T system, high-resolution images were acquired using an adiabatic spin-echo imaging sequence (repetition time [TR] 350 s, echo time [TE] 16.7 ms, number of acquisitions [NA] 16, sweep width 61728 Hz, matrix size 512×512, slice thickness 620 μm, field of view 12×12 mm^2^, 54 min/image) [33]. On the 7 T system, partial saturation T_1_ data were acquired (TE 13, matrix size 160×320, slice thickness 600 μm, field of view of either 7×7 mm^2^ [P14 and P20] or 8×8 mm^2^ [P50]). At each TR, several single images (number of images collected for each TR given in parenthesis) were acquired in the following order: TR 0.15 s (6), 3.5 s (1), 1.0 s (2), 1.9 s (1), 0.35 s (4), 2.7 s (1), 0.25 s (5) 0.5 s (3). These acquisition conditions provided 23.4–25 μm resolution across the central retina.

### Magnetic resonance imaging data analysis

Data from the 4.7 T system were analyzed as follows: Central retinal signal intensities were first extracted from each image using the program NIH IMAGE and derived macros [34], and the results from that group were compared with a generalized estimating equation approach (described below) [18]. Changes in receiver gain between animals were controlled for by setting the signal intensity of a fixed region of noise in each rat to a fixed value. Postreceptor (or inner retina [IR]) and receptor (or outer retina [OR]) signal intensity data (from 0.4 to 1 mm from the center of the optic nerve) were extracted as follows. As we have previously discussed, under these conditions, the IR/OR division is not observable in dark-adapted retinas [18]. To ensure that we were measuring from the IR and OR region, three pixels posterior to the retina/vitreous border and four pixels anterior to the retina/sclera border (both borders are easily observed) were analyzed to sample the IR and OR, respectively, as previously described [18].

Data from the 7 T system were analyzed as follows: Single images acquired with the same TR were first registered (rigid body), then averaged. These averaged images were then registered across TRs. The same regions-of-interest as above were analyzed by calculating 1/T_1_ maps by first fitting to a three-parameter T_1_ equation (y=a + b*(exp(-c*TR), where a, b, and c are fitted parameters) on a pixel-by-pixel basis using R (v.2.9.0, 163 R Development Core Team, 2009. R: A language and environment for statistical 164 computing. R Foundation for Statistical Computing, Vienna, Austria. ISBN 3–900051–165 07–0) scripts developed in house, and the minpack.lm package (v.1.1.1, Timur V. Elzhov and Katharine M. Mullen minpack.lm: R interface to the Levenberg-Marquardt nonlinear least-squares algorithm found in MINPACK. R package version 1.1–1.). The reciprocal (1/T_1_) maps directly reflect manganese levels [35]. These developmental data were reduced by the average baseline 1/T_1_ for LE rats (0.65 s^−1^) before calculating the percent change from age-matched controls.

To measure retinal thickness, we used in-house written software to first map the in situ image into a linear representation for each retina, as described previously [36]. First, the vitreoretinal border and optic nerve were manually defined. Using the center point of each highlighted pixel, a straight line was fit to the optic nerve, and a high-order polynomial (≤10th order) was fit to the vitreoretinal border. The intercept between the vitreoretinal border and the optic nerve lines served as the origin of the linearized image. Along the polynomial, roughly 10,000 evenly spaced points were then chosen by the program, and distances between each point and the one adjacent were calculated. Using these fine-grained linear approximations of distances along the polynomial, the program selects a line perpendicular to the polynomial every fifth of a pixel width (i.e., given resolutions of 23.4 or 25 μm, every 4.68 or every 5 μm, respectively) as measured along the polynomial. Intensity values along these lines were extracted and reconstructed into the linearized image. The linearized data from each hemiretina at 0.4–1 mm from the optic nerve were binned. For each bin, the average profile of signal intensity as a function of retinal depth was calculated, and the vitreous-retina and retina-choroid borders were found using the “half height” method [37]; the distance between these two borders is the whole retinal thickness.

### Developmental curve fitting

Developmental central retinal data for each strain (i.e., MEMRI and literature rhodopsin concentrations [38]) were fit to a Gompertz curve t (y=a*exp(-b*exp(-c*t)), where a (the upper limit), b (offset term=starting rate/c), and c (daily rate of growth or slope) are fitted parameters, and t is time in postnatal days [39]. The age at the inflection point (the point on the curve at which the sign of the curvature changes) is derived from the equation ln(b)/c. This fitting strategy was used to avoid making symmetry assumptions about the inflection point [39]. Only within-age averages were available when fitting data from the literature, and these were compared to the within-age means of the MEMRI data.

### Visual performance using optokinetic tracking

OKT was performed in control LE rats aged P32–36, and in VO LE rats between 14/19 and 14/26. The OKT stimulus conditions/parameters used to measure spatial frequency thresholds and contrast sensitivity curves have been described in detail previously [19,40]. In brief, a vertical sine wave grating (100% contrast) was projected as a virtual cylinder in three-dimensional coordinate space on computer monitors arranged in a quadrangle around a testing arena (OptoMotry; CerebralMechanics, Lethbridge, Alberta, Canada). Unrestrained rats (and not overnight dark-adapted ones) were placed on an elevated platform at the center of the arena. An experimenter used a video image of the arena from above to view the animal and follow the position of its head with the aid of a computer mouse and a crosshair superimposed on the frame. The X–Y positional coordinates of the crosshair centered the hub of the virtual cylinder, enabling its wall to be maintained at a constant apparent distance from the animal’s eyes. In this way, the spatial frequency of the stimulus was fixed at the animal’s viewing position, identical in all directions of gaze. When the cylinder was rotated in the clockwise or counter-clockwise direction and the animal followed with head and neck movements that tracked the rotation, it was judged that the animal could see the grating. For each animal, the highest spatial frequency that elicited a response was found, and this was considered the animal’s spatial frequency threshold. Contrast sensitivity was also evaluated at a preselected set of six spatial frequencies. Note that SD rats were not examined in this study because at baseline, they have a small OKT response, making them difficult to evaluate; therefore, examining them would have been nonideal for investigating reductions in visual performance associated with the 50/10 procedure [40].

### Statistical analysis

To compare the NV severity (in clock hours), a two-sample Mann–Whitney rank sum test (two-sided) was used because the severity scale used is limited to whole numbers. Sampling size (“n”) is the number of individual pups per experimental group. Individual pups from any given litter were assigned to the same experimental group and so, because groups were culled from either single or multiple litters, the possibility exists that litter effects could have influenced the main outcome variables. Neither experimental nor statistical tests for litter effects were conducted as suggested by Casella and others [41-43].

MEMRI data are presented as the mean and standard error of the mean (SEM) calculated from the mean data of each animal in that group. However, adjacent pixels within subject were correlated and the MEMRI data need to be compared using a generalized estimating equation (GEE) approach [18,44]. GEE performs a general linear regression analysis using all of the pixels in each subject and accounts for the within-subject correlation between adjacent pixels. GEE was performed using the GENMOD procedure in SAS for windows with the working correlation matrix set to autoregressive [1] and the scale parameter set to the Pearson chi-square.

Group differences in age- and strain-related changes in retinal thickness were assessed with ANCOVA (ANCOVA; age X group) analyses. The effect of diltiazem on retinal thickness was determined using a two-tailed *t* test comparison with age-matched controls.

Overlap between the 95% confidence intervals of the fitted Gompertz estimates was used to assess statistical significance for the derived inflection points and slopes. In all cases, two-tailed p<0.05 was considered statistically significant, unless otherwise noted. Data are presented as mean±SEM.

The OKT spatial frequency thresholds were consistent with a normal distribution and were analyzed using a two-tailed Students *t*-test; contrast sensitivity curves were analyzed using a two-way mixed ANOVA (group X spatial frequency) and post-hoc *t* tests at individual spatial frequencies. Data are presented as mean±SEM. A p<0.05 was considered statistically significant.

## Results

### Intraretinal manganese uptake in adult controls

In the dark-adapted P50 LE rat retina, intraretinal manganese uptake in uninjected (IR 0.82±0.05 s^−1^, OR 1.05±0.10 s^−1^, n=7, Table 1), saline-injected (IR 0.91±0.07 s^−1^, OR 1.04±0.08 s^−1^, n=3), or DMSO-injected (IR 0.86±0.03 s^−1^, OR 1.10±0.05 s^−1^, n=14) retinas were not different (p>0.05); these data were combined for further comparisons. As expected from previous light/dark adaptation studies in SD rats and C57Bl/6 mice, in LE controls, manganese levels were significantly (p<0.05) elevated in OR with dark adaption (1.08±0.04 s^−1^, n=24) compared to that in uninjected light-adapted rats (0.87±0.05 s^−1^, n=3). IR uptake did not change with dark (0.86±0.03 s^−1^, n=24) or light (0.84±0.10 s^−1^, n=3) adaption [17,18,23]. In addition, no strain differences (p<0.05) were found in the intraretinal uptake of dark-adapted P50 groups (Table 1) or during normal development (see below).

**Table 1:** Summary of Intraretinal Manganese Uptake (mean±SEM [n]).

**Group**	**Time point**			
**Controls**	**P14**	**P20**	**P50**	**Measure**
LE IR	0.83 ±0.03 [6]	0.85±0.03 [6]	0.82±0.05 [7]	1/T_1_ (sec^−1^)
LE OR	0.81±0.03	0.95±0.06	1.05±0.10	1/T_1_ (sec^−1^)
SD IR			0.87±0.03 [5]	1/T_1_ (sec^−1^)
SD OR			1.04±0.11	1/T_1_ (sec^−1^)
SD IR	71.5±2.6 [6]	75.7±2.5 [6]	82.2±2.6 [12]	SI (a.u.)a
SD OR	65.5±1.6	74.2±1.6	83.8±2.7	SI (a.u.)a
VO	14/0	14/6	14/34	
LE IR	0.94±0.03 [8]	1.00±0.02 [7]	1.12 ±0.12 [4]	1/T_1_ (sec^−1^)
LE OR	0.88±0.03	1.22±0.04	1.50±0.15	1/T_1_ (sec^−1^)
LE+DIL IR	–	0.89±0.06 [8]	–	1/T_1_ (sec^−1^)
LE+DIL OR	–	1.04±0.06	–	1/T_1_ (sec^−1^)
SD IR	87.3±2.2 [16]	73.7±2.4 [9]	69.1 ±2.3 [5]	SI (a.u.)a
SD OR	75.8±1.8	69.7 ±2.9	67.2±0.8	SI (a.u.)a

a: SI=signal intensity in arbitrary units (a.u.)

**Figure 1:**
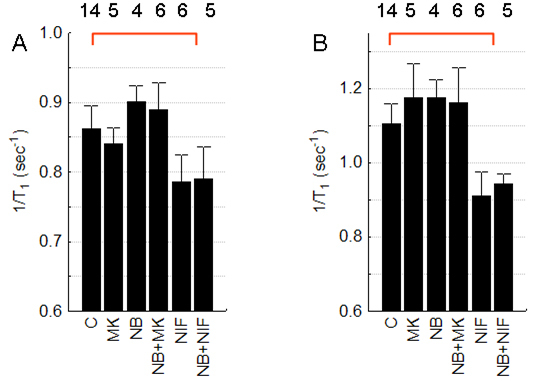
Summary of antagonists on intraretinal manganese uptake. Systemically administered antagonists were N-Methyl-D-aspartate (NMDA) receptor (MK-801 [MK]), 2-amino-3-(5-methyl-3-oxo-1,2- oxazol-4-yl)propanoic acid (AMPA) receptor (3-dihydroxy-6-nitro-7- sulfamoyl-benzo[f]quinoxaline-2,3-dione [NBQX; NB]), and L-type voltage-gated calcium channel (nifedipine (NIF)). Graphs depict **A**: Inner retina (IR) and **B**: Outer retina (OR) of dark adapted postnatal (P)50 Long Evans (LE) rats. The red line indicates a significant (p<0.05) difference from control values. Error bars represent standard error of the mean (SEM), and numbers above the bars represent the number of animals studied.

We also tested the effect on intraretinal manganese uptake of specific antagonists for L-type voltage-gated calcium channels (NIF), N-Methyl-D-aspartate (NMDA) receptors (MK), and 2-amino-3-(5-methyl-3-oxo-1,2- oxazol-4-yl)propanoic acid (AMPA) receptors (NB) in dark-adapted P50 LE retinas. Regardless of the combination of drugs systemically administered, only NIF significantly (p<0.05) reduced OR uptake to control light-adapted values (Figure 1). In IR, only NIF treatment reduced manganese uptake (p<0.05; Figure 1).

### Intraretinal manganese uptake during normal development

In control dark-adapted LE and SD rats, central OR manganese uptake significantly (p<0.05) increased from P14 to P50 (Table 2); during this period, IR uptake did not change in LE rats (p>0.05), but did in the SD group (p<0.05; Table 1). We next compared the developmental MEMRI time course in OR to previously reported strain-specific rhodopsin concentration data (percent change from P50 values): Similar slopes (overlapping 95% confidence intervals, Gompertz fitting) and inflection points were found regardless of strain (Table 2) [17,38]. The analysis in Table 2 is consistent with strain-independent uptake in central OR; no strain difference (p>0.05) OR or IR uptake (after converting to percent change from P50 values to account for the different types acquisition used in each group) was noted at P14 and P20 (data not shown).

**Table 2:** Growth Curve Gompertz Analysis.

**Growth curve group**	**Estimate**	**95% Conf. interval**	**Inflection point (PN days)**
**LE Rhodopsin [**38**]**			
a (upper limit)	105.63	71.24–140.00	11.9
b (offset)	2.59	−0. 46–5.64	
c (slope)	0.08	−0.01–0.18	
**SD Rhodopsin [**38**]**			
a (upper limit)	100.1	93.30–106.89	12.1
b (offset)	4.79	2.78–6.80	
c (slope)	0.13	0.10–0.17	
**LE MEMRI**			
a (upper limit)	102.13	a	13.7
b (offset)	4.51	a	
c (slope)	0.11	a	
**SD MEMRI**			
a (upper limit)	98.71	a	12.3
b (offset)	5.62	a	
c (slope)	0.14	a	

a: Exact fit to three time points so no confidence intervals

### Intraretinal manganese uptake following variable oxygen administration

**Figure 2:**
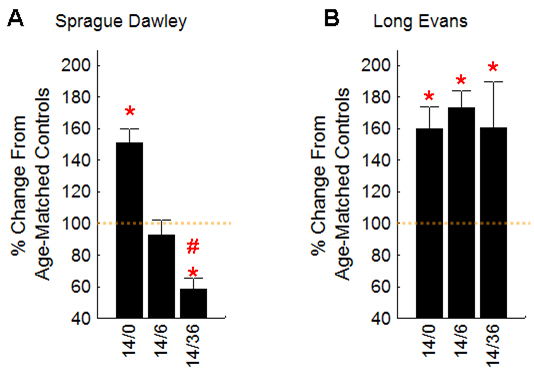
Summary of intraretinal manganese uptake time course data. Data are presented for outer retina as a percent change from age-controls (dotted orange line) for **A**: Sprague Dawley (SD) rats and **B**: variable oxygen (VO) rats. Inner retinal (IR) patterns were similar to these (outer retina) OR patterns. See Table 1 for numbers of rats investigated (n’s). Percent change from mean control and variable oxygen (VO) groups were calculated after subtraction of mean nonmanganese baseline values (50 arbitrary units (a.u.) for 4.7 T data and 0.65 s^−1^ for 7 T data). Error bars represent the SEM of only the VO animals. The * indicate a significant (p<0.05) difference from control values; the # indicates a significant (p<0.05) difference from the values at 14/0.

Following VO, the LE group had an IR and OR uptake increase between 14/0 and 14/36 (p<0.05), but in contrast, in the SD group, the extent of manganese accumulation decreased over this time (p<0.05). We summarized these data as a percent change from strain- and age-controls (Figure 2). Note that immediately after removal to room air at 14/0, the percent supernormal change for intraretinal uptake was not different (p>0.05) between LE and SD groups.

### Structural outcomes

#### Neovascularization

In room-air control P20 LE (data not shown) and SD rats [45], no retinal NV was noted. Following VO, NV severity in uninjected LE rats (10.3 clock hours [8-12]) was not different (p>0.05) from that in LE rats injected with saline between 7/0 and 14/6 (mean 10.1 clock hours [range 8.5–12]), and these data were combined for further comparisons. NV incidence was 100% in both VO LE (20/20) and SD (30/30) groups. NV severity was greater (p<0.05) in VO LE rats (10.2 [8.0–12.0]) relative to that in the VO SD group (7.4 [3.0–12.0]).

#### Thickness

Between P14 and P50, control central retinal thickness (Table 3) decreased (r=-0.94 [LE] and −0.89 [SD], p<0.05 [both groups], linear regression) with similar slopes (p>0.05, ANCOVA) but different intercepts (p<0.05, ANCOVA) between strains. VO rats also showed retinal thinning in that age range (r=-0.84 [LE] and −0.81 [SD], p<0.05 [both groups]) with similar (p>0.05) slopes and intercepts between strains. Relative to strain-specific controls, VO induced retinal thinning, as demonstrated by similar (p>0.05) slopes but different (p<0.05) intercepts. In VO LE rats, no differences in retinal thickness between untreated and diltiazem-treated rats were noted.

**Table 3:** Summary of Retinal Thickness (mean±SEM (n))

	**Retinal thickness (μm)**		
**Group**	**P14**	**P20**	**P50**
LE con	254±4 [8]	240±4 [6]	195±3 [7]
LE VO	216±9 [8]	212±5 [7]	153±6 [6]
LE VO±DIL	–	201±2 [8]	–
SD con	246±3 [5]	218±2 [6]	187±6 [5]
SD VO	229±5 [14]	212±4 [9]	168±4 [5]

### Visual performance

**Figure 3:**
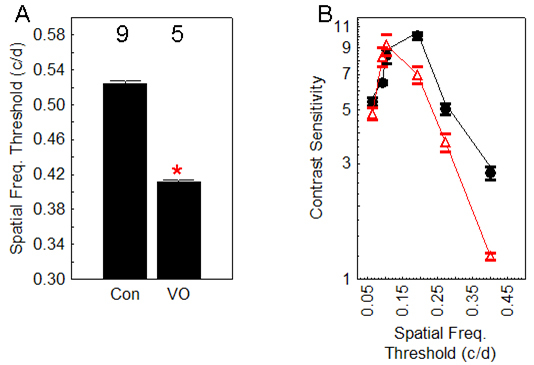
Summary of visual performance in variable oxygen exposed Long Evans rats. **A**: Spatial frequency threshold in control (con) and variable oxygen (VO) rats. The numbers above the bars represent the number of animals in each group. Error bars represent the standard error of the mean (SEM). The * indicates a significant (p<0.05) difference from control values. **B**: Contrast sensitivity in same two groups as **A**; circle=con, triangle=VO. These two curves are significantly different (ANOVA, p<0.05).

Because the retinas of VO LE rats experience retinal thinning and persistent supernormal intraretinal uptake compared with age-matched controls, we investigated the possibility that visual performance (i.e., spatial frequency threshold and contrast sensitivity) might also be affected. As shown in Figure 3, VO LE rats (n=5) had significantly (p<0.05) subnormal visual performance metrics relative to controls (n=9).

### Calcium channel antagonist treatment of variable oxygen rats

**Figure 4:**
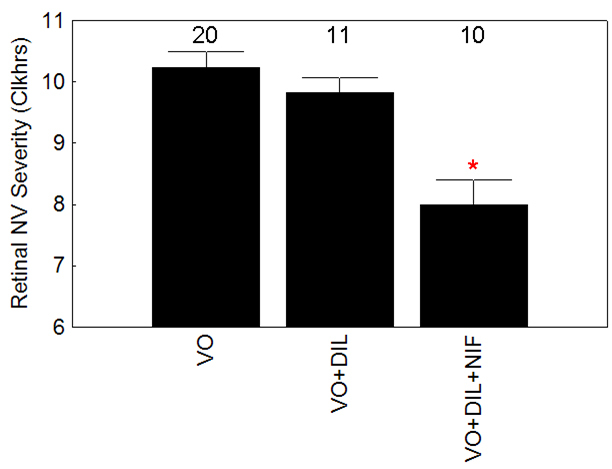
Summary of the severity retinal neovascularization (NV) severity in untreated variable oxygen (VO) Long Evans rats (VO), VO LE rats treated with diltiazem treatment (DIL) between 14/0 and 14/6 (VO+DIL), and VO LE rats treated with DIL+nifedipine (NIF) between 7/0 and 14/6 (VO+DIL+NIF). The * indicates a significant (p<0.05) difference from values in the VO group. Error bars represent standard error of the mean (SEM), and numbers above the bars represent the number of animals studied.

In treatment *arm A*, DIL administered to LE rats between 14/0 and 14/6 corrected (p<0.05) the supernormal intraretinal manganese uptake at P20 (Table 2) but not (p>0.05) retinal thinning (201.2±2 mm, n=8, Table 1) or NV severity (Figure 4). Next, we examined LE rats in treatment *arm B*, using a combination of DIL (30 mg/kg, s.c., between 7/0 and 14/6) and a chow admix to dam of NIF (between 7/0 and 14/6) (DIL+NIF) verses saline (s.c, between 7/0 and 14/6, control) and normal chow (7/0 and 14/6). This combination of DIL+NIF administered during and after VO significantly reduced (p<0.05) NV severity (8 clock hours [6-10]) by 28% (p<0.05) compared to that of the saline-treated and nontreated control groups (Figure 4). In all cases, NV incidence was 100%.

## Discussion

In this study, we found that intraretinal manganese uptake in adult control LE rats was principally regulated by L-type voltage-gated calcium channels [4,13,46,47]. The supernormal uptake of manganese following VO suggested a dramatic increase in intraretinal calcium channel activity in both the LE and SD groups. Indeed, specific calcium channel inhibition applied after VO could normalize this supernormal uptake, and importantly, when applied both during and after VO, significantly reduced retinal NV severity. The exact mechanism(s) by which the combination of DIL+NIF suppressed NV was not investigated in this study. It is possible that non-L-type calcium channel effects of diltiazem contributed to the observed effect. However, we note that the DIL+NIF combination may antagonize L-type channels synergistically [48]. Although we have shown that both drugs will inhibit retinal Mn^2+^ uptake (this study) [4],we did not measure DIL or NIF retinal concentrations in this study and so cannot address whether or not each drug reached the saturation level for its target. In addition, systemic calcium channel blockers can have both a systemic influence (on, for example, blood pressure) and local effects on the retina. Nonetheless, these data suggest that additional NV inhibition might be achieved using different drug combinations, doses, routes, and/or treatment schedules. These data are consistent with a previous report of supernormal intraretinal levels of calcium, but not sodium, potassium, or magnesium ions, in another newborn rodent retinal NV model [11]. The above considerations strongly support a role for intraretinal calcium channels in retinal NV in a VO model of ROP.

The responses to VO in LE and SD rats could not have been predicted based on baseline measures of retinal structure and function: Control groups of both strains had similar patterns of these metrics during and after retinal maturation (e.g., Table 1, Table 2, and Table 3). The importance of the slightly thicker retinas in LE (by 5.5%) than those of the SD group in vivo is not clear and no such strain thickness differences seem present histologically [49]. MEMRI functional findings in controls are consistent with ERG results showing similar retinal function in scotopic conditions in pigmented and albino rat retina [50]. Previously, we demonstrated in adult rats that the visual cycle can regulate OR uptake of manganese [17]. In this study, we extend these findings to young rats by showing agreement between OR uptake of manganese and published rhodopsin concentrations. The agreement between these results is encouraging, since the rhodopsin levels were collated from data in several laboratories, and sampled at somewhat different postnatal time points.

The drugs used herein were chosen to modulate specific retinal calcium channels in vivo. Two classes of voltage-gated calcium channel blockers were investigated: the benzothiazepine DIL, which primarily antagonizes L-type voltage-gated calcium channels in vivo (although DIL has been found to weakly inhibit the sodium/calcium exchanger and cyclic guanosine monophosphate (cGMP)-activated conductance in vitro [26-29]), and the somewhat more L-type specific dihydropyridine NIF [51,52]; both drugs are effective in the retina in vivo on MEMRI examination (this study) [4]. In addition, we used MK, a specific antagonist for the NMDA receptor in the retina [14,53], and NB, a specific antagonist for AMPA receptors in the retina [54]; both inhibitors strongly affect behavior, indicating passage through the blood-brain barrier [55,56]. The present MK and NB results appear consistent with a complex stimulatory and inhibitory regulation of IR neuronal activity involving NMDA and AMPA receptors, and the reported lack of such regulation in the OR [57].

In addition to NV, we found evidence for VO-induced abnormal dark adaptation, raising the possibility that nonvascular retinal function was also compromised. Previously, we reported a supernormal intraretinal uptake measured in light-adapted VO SD rats at 14/6 [4], whereas at the 14/6 time point in the present study, uptake in the IR and OR of the dark-adapted VO SD group was normal. Since, in controls, OR uptake is higher in dark-adapted rodents than in light-adapted conditions (this study) [18,58], it appears that VO substantially altered the normal light/dark adaptation uptake patterns, and thus possibly reduced the visual processing ability of the central retina. To investigate this idea, we examined the visual performance of control and VO LE rats using OKT. In control LE rats, our spatial frequency threshold (0.52±0.008 c/d) was similar to that published by Douglas et al. [40] (0.54±0.0013 c/d), although a different contrast sensitivity curve was found. The reason for this discrepancy in the contrast curves is not clear [59]. Nonetheless, within-laboratory comparisons were likely valid. Using the same OKT protocol, we found that VO reduced both spatial frequency threshold and contrast sensitivity. Visual performance was not measured in SD rats because their baseline OKT response is much more difficult to discern than that of pigmented animals (like LE), and thus, in general, detection sensitivity is much lower [40]. Note that OKT is influenced by both retinal and postretinal processing. This appears to explain why albino rats, with their abnormal optic nerve chiasm crossing relative to that in pigmented rats [50,60,61], have relatively poorer OKT responses, even though subtle-to-no-baseline retinal-specific differences are noted on electroretinography (ERG) and MEMRI between strains [50]. In other words, the real-life consequences of visual system abnormalities are not clear without some measure of visual performance. On the other hand, metrics of visual performance, such as OKT, provide insufficient information for evaluating retinal-specific deficits. These considerations strongly suggest that the combination of OKT and MEMRI should be used to fully appreciate the impact of disease on vision. Alternative functional evaluation, such as ERG, can also be used, although ERG does not specifically measure central retinal function and cannot be colocalized with regional changes in retinal thickness. We also note that once each strain was placed back into room air, a dramatically different temporal evolution of the retinal uptake patterns occurred. We speculate that genetic differences dominated retinal ion regulation following VO during room-air exposure. These observations are reminiscent of clinical reports of a possible genetic foundation to ROP morbidity [62-64]. The above considerations strongly imply that VO produces not only vascular morbidity (NV) but also important nonvascular functional lesions (abnormal dark adaptation and visual performance).

An additional explanation for the reduction in vision may involve the significant degree of retinal thinning we measured following VO, relative to age-matched controls. Central retinal thinning has been previously noted at 14/6 in VO LE and SD rats [4,7,65]. In this study, we find significant pathological thinning (in contrast to normal age-related thinning) by P14 in both strains. However, no further thinning was noted once the animals were in room air, suggesting that neuroprotective interventions from 14/0 onward would be ineffective. Indeed, diltiazem administered between 14/0 and 14/6 did not prevent pathological retinal thinning in VO rats. Our data raise the possibility that experimental ROP retinal thinning and supernormal manganese uptake comprise leading indicators of retinal morbidity, and that retinal NV is a lagging indicator. It is not yet clear what combination of thinning and impaired function measured in the VO groups is sufficient to reduce visual behavior.

In this study, we found evidence for early and sustained retinal structural and functional abnormalities, severe NV, and impaired visual performance in LE rats following the VO procedure. Importantly, specific L-type calcium channel antagonism, which can reduce central supernormal intraretinal manganese uptake, reduced retinal NV. Even assuming a clinically achievable reduction of NV by 28%, the present experimental results cannot predict how such a reduction would alter visual outcomes. Future studies are envisioned that investigate the efficacy of topically administered calcium channel antagonists. While retinal and vision abnormalities linked with extreme prematurity likely do not have a single biochemical etiology, the design of combination therapies in the future will benefit from an understanding of whether or not to include L-type calcium channel modulators. Nevertheless, these data, together with those in the literature [4,9-11], justify further investigations into changes in calcium ion regulation during and after VO that seem linked to retinal NV, structural and functional anomalies, and vision loss in ROP.

## References

[1] O'Connor A, Fielder AR (2008). Long term ophthalmic sequelae of prematurity.. Early Hum Dev.

[2] Dorfman A, Dembinska O, Chemtob S, Lachapelle P (2008). Early manifestations of postnatal hyperoxia on the retinal structure and function of the neonatal rat.. Invest Ophthalmol Vis Sci.

[3] Liu K, Akula JD, Falk C, Hansen RM, Fulton AB (2006). The retinal vasculature and function of the neural retina in a rat model of retinopathy of prematurity.. Invest Ophthalmol Vis Sci.

[4] Berkowitz BA, Roberts R, Penn JS, Gradianu M (2007). High-resolution manganese-enhanced MRI of experimental retinopathy of prematurity.. Invest Ophthalmol Vis Sci.

[5] Barnaby AM, Hansen RM, Moskowitz A, Fulton AB (2007). Development of scotopic visual thresholds in retinopathy of prematurity.. Invest Ophthalmol Vis Sci.

[6] Fulton AB, Hansen RM, Moskowitz A, Barnaby AM (2005). Multifocal ERG in subjects with a history of retinopathy of prematurity.. Doc Ophthalmol.

[7] Akula JD, Favazza TL, Mocko JA, Benador IY, Asturias AL, Kleinman MS, Hansen RM, Fulton AB (2010). The anatomy of the rat eye with oxygen-induced retinopathy.. Documenta Ophthalmologica..

[8] Harris ME, Moskowitz A, Fulton A, Hansen R (2011). Long-term effects of retinopathy of prematurity (ROP) on rod and rod-driven function.. Doc Ophthalmol.

[9] Juárez CP, Muiño JC, Guglielmone H, Sambuelli R, Echenique JR, Hernández M, Luna JD (2000). Experimental retinopathy of prematurity: angiostatic inhibition by nimodipine, ginkgo-biloba, and dipyridamole, and response to different growth factors.. Eur J Ophthalmol.

[10] Higgins RD, Yu K, Sanders RJ, Nandgaonkar BN, Rotschild T, Rifkin DB (1999). Diltiazem reduces retinal neovascularization in a mouse model of oxygen induced retinopathy.. Curr Eye Res.

[11] Kim JH, Kim JH, Yu YS, Kim DH, Lee TG, Moon DW, Kim KW (2008). In situ calcium mapping in the mouse retina via time-of-flight secondary ion mass spectrometry: modulation of retinal angiogenesis by calcium ion in development and oxygen-induced retinopathy.. Biochem Cell Biol.

[12] Ahlijanian MK, Westenbroek RE, Catterall WA (1990). Subunit structure and localization of dihydropyridine-sensitive calcium channels in mammalian brain, spinal cord, and retina.. Neuron.

[13] Drapeau P, Nachshen DA (1984). Manganese fluxes and manganese-dependent neurotransmitter release in presynaptic nerve endings isolated from rat brain.. J Physiol.

[14] Melena J, Osborne NN (2001). Voltage-dependent calcium channels in the rat retina: involvement in NMDA-stimulated influx of calcium.. Exp Eye Res.

[15] Barnett JM, Yanni S, Penn J (2010). The development of the rat model of retinopathy of prematurity.. Documenta Ophthalmologica..

[16] Berkowitz BA, Gradianu M, Schafer S, Jin Y, Porchia A, Iezzi R, Roberts R (2008). Ionic dysregulatory phenotyping of pathologic retinal thinning with manganese-enhanced MRI.. Invest Ophthalmol Vis Sci.

[17] Berkowitz BA, Roberts R, Oleske DA, Chang M, Schafer S, Bissig D, Gradianu M (2009). Quantitative mapping of ion channel regulation by visual cycle activity in rodent photoreceptors in vivo.. Invest Ophthalmol Vis Sci.

[18] Berkowitz BA, Roberts R, Goebel DJ, Luan H (2006). Noninvasive and simultaneous imaging of layer-specific retinal functional adaptation by manganese-enhanced MRI.. Invest Ophthalmol Vis Sci.

[19] Prusky GT, Alam NM, Beekman S, Douglas RM (2004). Rapid quantification of adult and developing mouse spatial vision using a virtual optomotor system.. Invest Ophthalmol Vis Sci.

[20] Umino Y, Solessio E, Barlow RB (2008). Speed, spatial, and temporal tuning of rod and cone vision in mouse.. J Neurosci.

[21] Cahill H, Nathans J (2008). The optokinetic reflex as a tool for quantitative analyses of nervous system function in mice: application to genetic and drug-induced variation.. PLoS ONE.

[22] Prusky GT, Silver BD, Tschetter WW, Alam NM, Douglas RM (2008). Experience-dependent plasticity from eye opening enables lasting, visual cortex-dependent enhancement of motion vision.. J Neurosci.

[23] Berkowitz BA, Gradianu M, Bissig D, Kern TS, Roberts R (2009). Retinal ion regulation in a mouse model of diabetic retinopathy: Natural history and the effect of Cu/Zn superoxide dismutase overexpression.. Invest Ophthalmol Vis Sci.

[24] Berkowitz BA, Penn JS (1998). Abnormal panretinal response pattern to carbogen inhalation in experimental retinopathy of prematurity.. Invest Ophthalmol Vis Sci.

[25] Penn JS, Henry MM, Wall PT, Tolman BL (1995). The range of PaO2 variation determines the severity of oxygen-induced retinopathy in newborn rats.. Invest Ophthalmol Vis Sci.

[26] Hart J, Wilkinson MF, Kelly MEM, Barnes S (2003). Inhibitory action of diltiazem on voltage-gated calcium channels in cone photoreceptors.. Exp Eye Res.

[27] Cox DA, Conforti L, Sperelakis N, Matlib MA (1993). Selectivity of inhibition of Na(+)-Ca2+ exchange of heart mitochondria by benzothiazepine CGP-37157.. J Cardiovasc Pharmacol.

[28] Stern JH, Kaupp UB, Macleish PR (1986). Control of the light-regulated current in rod photoreceptors by cyclic GMP, calcium, and l–cis-diltiazem.. Proc Natl Acad Sci USA.

[29] Koch KW, Kaupp UB (1985). Cyclic GMP directly regulates a cation conductance in membranes of bovine rods by a cooperative mechanism.. J Biol Chem.

[30] Lutty GA, McLeod DS (1992). A new technique for visualization of the human retinal vasculature.. Arch Ophthalmol.

[31] Berkowitz BA, Roberts R (2010). Evidence for a critical role of panretinal pathophysiology in experimental ROP.. Documenta Ophthalmologica..

[32] Zhang S, Leske DA, Holmes JM (2000). Neovascularization grading methods in a rat model of retinopathy of prematurity.. Invest Ophthalmol Vis Sci.

[33] Schupp DG, Merkle H, Ellermann JM, Ke Y, Garwood M (1993). Localized detection of glioma glycolysis using edited 1H MRS.. Magn Reson Med.

[34] Berkowitz BA (1996). Adult and newborn rat inner retinal oxygenation during carbogen and 100% oxygen breathing. Comparison using magnetic resonance imaging delta Po2 mapping.. Invest Ophthalmol Vis Sci.

[35] Chuang KH, Koretsky AP, Sotak CH (2009). Temporal changes in the T1 and T2 relaxation rates (DeltaR1 and DeltaR2) in the rat brain are consistent with the tissue-clearance rates of elemental manganese.. Magn Reson Med.

[36] Berkowitz BA, Roberts R, Luan H, Bissig D, Bui BV, Gradianu M, Calkins DJ, Vingrys AJ (2007). Manganese-enhanced MRI studies of alterations of intraretinal ion demand in models of ocular injury.. Invest Ophthalmol Vis Sci.

[37] Cheng H, Nair G, Walker TA, Kim MK, Pardue MT, Thulé PM, Olson DE, Duong TQ (2006). Structural and functional MRI reveals multiple retinal layers.. Proc Natl Acad Sci USA.

[38] Timmers AM, Fox DA, He L, Hansen RM, Fulton AB (1999). Rod photoreceptor maturation does not vary with retinal eccentricity in mammalian retina.. Curr Eye Res.

[39] Easton DM (2005). Gompertzian growth and decay: A powerful descriptive tool for neuroscience.. Physiol Behav.

[40] Douglas RM, Alam NM, Silver BD, McGill TJ, Tschetter WW, Prusky GT (2005). Independent visual threshold measurements in the two eyes of freely moving rats and mice using a virtual-reality optokinetic system.. Vis Neurosci.

[41] Casella G. Statistical inference. Duxbury/Thomson Learning; 2008.

[42] Scott S, Kranz JE, Cole J, Lincecum JM, Thompson K, Kelly N, Bostrom A, Theodoss J, Al-Nakhala BM, Vieira FG, Ramasubbu J, Heywood JA (2008). Design, power, and interpretation of studies in the standard murine model of ALS.. Amyotroph Lateral Scler.

[43] Hockly E, Woodman B, Mahal A, Lewis CM, Bates G (2003). Standardization and statistical approaches to therapeutic trials in the R6/2 mouse.. Brain Res Bull.

[44] Liang Z (1986). Longitudinal data analysis using generalized linear models.. Biometrika.

[45] Berkowitz BA, Zhang W (2000). Significant reduction of the panretinal oxygenation response after 28% supplemental oxygen recovery in experimental ROP.. Invest Ophthalmol Vis Sci.

[46] Matsushita K, Fukumoto M, Kobayashi T, Kobayashi M, Ishizaki E, Minami M, Katsumura K, Liao SD, Wu DM, Zhang T, Puro DG (2010). Diabetes-induced inhibition of voltage-dependent calcium channels in the retinal microvasculature: role of spermine.. Invest Ophthalmol Vis Sci.

[47] Lu H, Xi ZX, Gitajn L, Rea W, Yang Y, Stein EA (2007). Cocaine-induced brain activation detected by dynamic manganese-enhanced magnetic resonance imaging (MEMRI).. Proc Natl Acad Sci USA.

[48] Bellemann P, Schade A, Towart R (1983). Dihydropyridine receptor in rat brain labeled with [3H]nimodipine.. Proc Natl Acad Sci USA.

[49] Kirwin SJ, Kanaly ST, Linke NA, Edelman JL (2009). Strain-dependent increases in retinal inflammatory proteins and photoreceptor FGF-2 expression in streptozotocin-induced diabetic rats.. Invest Ophthalmol Vis Sci.

[50] Heiduschka P, Schraermeyer U (2008). Comparison of visual function in pigmented and albino rats by electroretinography and visual evoked potentials.. Graefes Arch Clin Exp Ophthalmol.

[51] Puro DG, Mano T (1991). Modulation of calcium channels in human retinal glial cells by basic fibroblast growth factor: a possible role in retinal pathobiology.. J Neurosci.

[52] Bean BP (1989). Classes of calcium channels in vertebrate cells.. Annu Rev Physiol.

[53] Itoh K, Sakata M, Watanabe M, Aikawa Y, Fujii H (2008). The entry of manganese ions into the brain is accelerated by the activation of N-methyl-d-aspartate receptors.. Neuroscience.

[54] Cohen ED, Miller RF (1999). The network-selective actions of quinoxalines on the neurocircuitry operations of the rabbit retina.. Brain Res.

[55] Colwell CS, Ralph MR, Menaker M (1990). Do NMDA receptors mediate the effects of light on circadian behavior?. Brain Res.

[56] Maeng S, Zarate CA, Du J, Schloesser RJ, McCammon J, Chen G, Manji HK (2008). Cellular mechanisms underlying the antidepressant effects of ketamine: Role of [alpha]-amino-3-hydroxy-5-methylisoxazole-4-propionic acid receptors.. Biol Psychiatry.

[57] Pourcho RG, Qin P, Goebel DJ (2001). Cellular and subcellular distribution of NMDA receptor subunit NR2B in the retina.. J Comp Neurol.

[58] Berkowitz BA, Roberts R, Oleske DA, Chang M, Schafer S, Bissig D, Gradianu M (2009). Quantitative mapping of ion channel regulation by visual cycle activity in rodent photoreceptors in vivo.. Invest Ophthalmol Vis Sci.

[59] Keller J, Strasburger H, Cerutti DT, Sabel BA (2000). Assessing spatial vision–automated measurement of the contrast-sensitivity function in the hooded rat.. J Neurosci Methods.

[60] Jeffery G (1997). The albino retina: an abnormality that provides insight into normal retinal development.. Trends Neurosci.

[61] Prusky GT, Harker KT, Douglas RM, Whishaw IQ (2002). Variation in visual acuity within pigmented, and between pigmented and albino rat strains.. Behav Brain Res.

[62] Bizzarro MJ, Hussain N, Jonsson B, Feng R, Ment LR, Gruen JR, Zhang H, Bhandari V (2006). Genetic susceptibility to retinopathy of prematurity.. Pediatrics.

[63] Aralikatti AK, Mitra A, Denniston AK, Haque MS, Ewer AK, Butler L (2010). Is ethnicity a risk factor for severe retinopathy of prematurity?. Arch Dis Child Fetal Neonatal Ed.

[64] Mohamed S, Schaa K, Cooper ME, Ahrens E, Alvarado A, Colaizy T, Marazita ML, Murray JC, Dagle JM (2009). Genetic contributions to the development of retinopathy of prematurity.. Pediatr Res.

[65] Dorfman AL, Polosa A, Joly S, Chemtob S, Lachapelle P (2009). Functional and structural changes resulting from strain differences in the rat model of oxygen-induced retinopathy.. Invest Ophthalmol Vis Sci.

